# Prediction of the clinicopathological subtypes of breast cancer using a fisher discriminant analysis model based on radiomic features of diffusion-weighted MRI

**DOI:** 10.1186/s12885-020-07557-y

**Published:** 2020-11-09

**Authors:** Ming Ni, Xiaoming Zhou, Jingwei Liu, Haiyang Yu, Yuanxiang Gao, Xuexi Zhang, Zhiming Li

**Affiliations:** 1 Department of Radiology, The Affiliated Hospital of Qingdao University, No.59 Haier Road, Qingdao, 266000 China; 2 Department of Pediatric Surgery, Shandong University Qilu Hospital, Jinan, 250012 China; 3 Life Science, GE Healthcare China, Shanghai, 201203 China

**Keywords:** Clinicopathological subtype, Fisher discriminant analysis, Diffusion-weighted imaging

## Abstract

**Background:**

The clinicopathological classification of breast cancer is proposed according to therapeutic purposes. It is simplified and can be conducted easily in clinical practice, and this subtyping undoubtedly contributes to the treatment selection of breast cancer. This study aims to investigate the feasibility of using a Fisher discriminant analysis model based on radiomic features of diffusion-weighted MRI for predicting the clinicopathological subtypes of breast cancer.

**Methods:**

Patients who underwent breast magnetic resonance imaging were confirmed by retrieving data from our institutional picture archiving and communication system (PACS) between March 2013 and September 2017. Five clinicopathological subtypes were determined based on the status of ER, PR, HER2 and Ki-67 from the immunohistochemical test. The radiomic features of diffusion-weighted imaging were derived from the volume of interest (VOI) of each tumour. Fisher discriminant analysis was performed for clinicopathological subtyping by using a backward selection method. To evaluate the diagnostic performance of the radiomic features, ROC analyses were performed to differentiate between immunohistochemical biomarker-positive and -negative groups.

**Results:**

A total of 84 radiomic features of four statistical methods were included after preprocessing. The overall accuracy for predicting the clinicopathological subtypes was 96.4% by Fisher discriminant analysis, and the weighted accuracy was 96.6%. For predicting diverse clinicopathological subtypes, the prediction accuracies ranged from 92 to 100%. According to the cross-validation, the overall accuracy of the model was 82.1%, and the accuracies of the model for predicting the luminal A, luminal B_HER2-_, luminal B_HER2+_, HER2 positive and triple negative subtypes were 79, 77, 88, 92 and 73%, respectively. According to the ROC analysis, the radiomic features had excellent performance in differentiating between different statuses of ER, PR, HER2 and Ki-67.

**Conclusions:**

The Fisher discriminant analysis model based on radiomic features of diffusion-weighted MRI is a reliable method for the prediction of clinicopathological breast cancer subtypes.

## Background

Breast cancer is the second most common cancer cause of cancer death in females [1]. Based on gene expression profiling, four intrinsic molecular subtypes can be defined: luminal A, luminal B, human epidermal growth factor receptor 2 (HER2)-enriched, and basal-like [2–4].

One clinicopathological classification of breast cancer focused on therapeutic purposes has been adopted by the 12th International Breast Cancer Conference [5]. These clinicopathological subtypes are similar but not identical to the intrinsic molecular subtypes. There are five clinicopathological subtypes including luminal A, luminal B_HER2-_ (luminal B/HER2 negative), luminal B_HER2+_ (luminal B/HER2 positive), HER2 positive and triple negative [5] (Table 1). Four immunohistochemical (IHC) biomarkers, including oestrogen receptor (ER), progesterone receptor (PR), HER2, and Ki-67, are recommended to define the clinicopathological subtypes. This classification is aimed at systematic therapy: luminal A cases require endocrine therapy; luminal B_HER2-_ cases require endocrine therapy with or without cytotoxic therapy; luminal B_HER2+_ cases require cytotoxic, anti-HER2 and endocrine therapy; HER2 positive cases require cytotoxic and anti-HER2 therapy; and triple negative cases require cytotoxic therapy. At least two advantages of the clinicopathological subtypes are as follows: first, in contrast to high cost and time-consuming gene expression array testing, clinicopathological subtyping is simplified and can be conducted easily in clinical practice; second, this subtyping undoubtedly contributes to the treatment selection of breast cancer.

**Table 1:** Clinicopathological Subtypes and Clinical Decision-Making [Ref [5]

Clinicopathological Subtype	IHC status	Clinical Decision-Making
**Luminal A**	ER and/or PR positive HER2 negative Ki-67 low (< 14%)	Endocrine therapy
^**a**^**Luminal B**_**HER2-**_	ER and/or PR positive HER2 negative Ki-67 high	Endocrine±cytotoxic therapy
^**b**^**Luminal B**_**HER2+**_	ER and/or PR positive any Ki-67 HER2 over-expressed	Cytotoxics + anti-HER2 + endocrine therapy
**HER2 positive**	HER2 over-expressed	Cytotoxics + anti-HER2
**Triple negative**	ER and PR absent HER2 negative	Cytotoxics

^a^*Luminal B*_*HER2-*_ luminal B/HER2 negative, ^b^*Luminal B*_*HER2+*_ luminal B/HER2 positive, *IHC* immunohistochemistry

Diffusion-weighted imaging (DWI) is an essential sequence that can monitor the mobility of water molecules. With restricted water diffusion, breast cancer usually shows hyperintensity on DW images [6]. DWI contributes to the differential diagnosis of breast lesions and may be a promising tool in breast cancer detection [7]. In differentiating malignant and benign breast lesions, the diagnostic performance of contrast-enhanced magnetic resonance imaging (MRI) with DWI is higher than that of contrast-enhanced MRI with time-intensity curves [8]. In addition, DWI also has the potential to monitor radiation-induced treatment response and neoadjuvant treatment response [9, 10]. More importantly, DWI can be an alternative for breast cancer screening without contrast media [6].

Radiomics is a process of converting digital medical images into mineable high-dimensional data [11]. It has been used in the detection and diagnosis of cancer, assessment of prognosis, prediction of response to treatment, and monitoring of disease status [11, 12]. The applications of radiomics in breast cancer include the prediction of molecular classification [13, 14], assessment of tumour recurrence [15], and response to treatment [16]. Recently, radiomics, by using the image phenotyping of breast cancers and their surrounding parenchyma on dynamic contrast-enhanced MRI, was used to identify triple-negative breast cancer [13]. Another radiomic study showed a positive trend between the molecular cancer subtype and breast tumour phenotype of size and enhancement texture based on dynamic contrast-enhanced (DCE) MRI [14]. Unfortunately, the differential diagnosis of diverse molecular subtypes was not explored in this study. Mammographic radiomic features could also be used for the prediction of breast cancer molecular subtypes with oversimplified classifications such as triple-negative and non-triple-negative, HER2-enriched and non-HER2-enriched, and luminal and non-luminal [17]. Radiogenomics is a novel approach that can correlate imaging characteristics with underlying genes, mutations and expression patterns at the genetic level [18]. Radiogenomics can be imaging surrogates for genetic tests and can reflect tumour biology [19]. One recent study demonstrated that radiogenomics of breast cancer could infer underlying gene expression by using RNA sequencing [20]. Breast tumours with higher expression levels of the JAK/STAT and VEGF pathways had more intratumour heterogeneity in image enhancement texture detected by using dynamic contrast-enhanced MRI. Fan et al. showed that the image features of DCE-MRI were associated with gene expression modules and could predict the prognosis of breast cancer patients [21]. In addition, for breast cancer subtyping, the combination of correlated miRNAs and imaging features has better classification power in differentiating luminal A and other breast cancer subtypes than using miRNAs or imaging alone [22]. By means of deep learning or novel algorithms, the breast cancer molecular subtypes were differentiated based on image features from DCE-MRI [23, 24].

As mentioned above, the clinicopathological subtypes of breast cancer are essential for treatment selection. Thus, it is urgent to develop a reliable method for the prediction of the clinicopathological subtypes. Diffusion-weighted imaging has high sensitivity in detecting breast cancer and is widely used in clinical practice. Herein, our study aims to verify the feasibility of using a radiomic approach based on DWI for the prediction of clinicopathological breast cancer subtypes.

## Methods

### Study population

This retrospective study was approved by our institutional ethics committee of The Affiliated Hospital of Qingdao University, and the informed consent was waived. A total of 112 patients that underwent breast MRI were confirmed by retrieving data from our institutional picture archiving and communication system (PACS) between March 2013 and September 2017. The inclusion criteria were as follows: (1) patients who had suspected breast tumours and underwent breast MRI; (2) patients with malignant breast tumours confirmed by histopathological examination; (3) patients with ER, PR, HER2 and Ki-67 status obtained from immunohistochemical analysis; and (4) high-quality DW images used for outlining the lesions, without a size threshold for the lesions. The exclusion criteria were as follows: (1) patients with breast lesions who underwent any treatment before breast MRI, including surgery, chemotherapy, radiotherapy, or anti-HER2 therapy; (2) patients with bilateral breast lesions; (3) patients with suspected metastatic breast tumours; (4) DW images were illegible for assessment; (5) patients with pseudotumours or tumour-like lesions, including chronic inflammatory nodules, adenosis of the breast, and fat necrosis nodules; and (6) patients with tumours located in the skin and areola.

### Clinicopathological subtyping

The immunohistochemical data of 112 patients were obtained by retrieval from the hospital information system. The statuses of ER, PR, HER2 and Ki-67 were determined by immunohistochemical tests. ER and PR expression were considered positive if at least 1% of tumour cells showed positive nuclear staining [25]. HER2 status was defined as positive if it presents an immunohistochemical score of 3+ and/or if in situ hybridization is positive [26]. A Ki-67 index higher than 14% is regarded as being at a high level [27]. There are five clinicopathological subtypes of breast cancers [5]: luminal A, ER and/or PR positive, HER2 negative and Ki-67 low (< 14%); luminal B_HER2-_ (luminal B/HER2 negative), ER and/or PR positive, HER2 negative and Ki-67 high; luminal B_HER2+_ (luminal B/HER2 positive), ER and/or PR positive, any Ki-67 and HER2 overexpressed or amplified; HER2 positive, HER2 overexpressed or amplified, ER and PR absent; triple negative, ER and PR absent, HER2 negative.

### Imaging data

All 112 patients underwent breast MR examinations on a 3.0 T MR system (MAGNETOM Skyra, Siemens Healthineers). Only diffusion-weighted MRI was used in this study. The DW imaging was performed with a 4-channel breast coil while the patients were in the prone position: axial imaging plane; repetition time/echo time, 5400/55 ms; field of view, 350 mm; voxel size, 1.8 × 1.8 × 5.0 mm; slice thickness 5 mm; spacing 0 mm; NEX 2; acquisition matrix 128 × 128; b value (s/mm^2^), 0 and 800. The acquisition time of DWI was approximately 125 s. Other imaging protocols were as follows: (1) axial T2-weighted imaging with fat-suppression/SPAIR: repetition time/echo time, 3500/68 ms; field of view, 350 mm; voxel size, 0.5 × 0.5 × 5.0 mm; slice thickness 5 mm; flip angle 80°; NEX 1; (2) sagittal T2-weighted imaging with fat-suppression: repetition time/echo time, 3200/66 ms; field of view, 260 mm; voxel size, 0.8 × 0.8 × 4.0 mm; slice thickness 4 mm; flip angle 120°; NEX 1; (3) axial T1-weighted imaging without fat-suppression: repetition time/echo time, 6/2.46 ms; field of view, 340 mm; voxel size, 0.8 × 0.8 × 1.6 mm; slice thickness 1.6 mm; flip angle 15°; NEX 1; (4) 3D T1-weighted pre-contrast imaging with fat-suppression: repetition time/echo time, 4.49/1.68 ms; field of view, 340 mm; voxel size, 1.0 × 1.0 × 1.2 mm; slice thickness 1.2 mm; flip angle 10°; NEX 1; and (5) 3D-DCE T1-weighted imaging with fat-suppression by injection of Gd-DTPA (0.1 mmol/kg), acquiring seven phases after injection. The entire acquisition time was approximately 26 min.

### Image segmentation and feature extraction

The diffusion-weighted images of each patient were saved and transferred to a radiomics analysis package, i.e., Artificial Intelligent Kit (A.K.) software (GE Healthcare, Shanghai, Version 3.0.1). The T2-weighted images and DCE-MR images were reviewed for lesion validation. The segmentation of breast tumours on DW images (b value, 800) was performed by using a two-step approach: first, the tumour margin was delineated manually slice by slice, and regions of interest (ROIs) were obtained; second, these ROIs were merged automatically by the A.K. software, and the volume of interest (VOI) of a tumour was finally completed. During ROI determination, both cystic and necrotic areas of the tumour were included in the ROI. Moreover, only the largest lesion was selected in patients with multiple unilateral tumours.

**Fig. 1:**
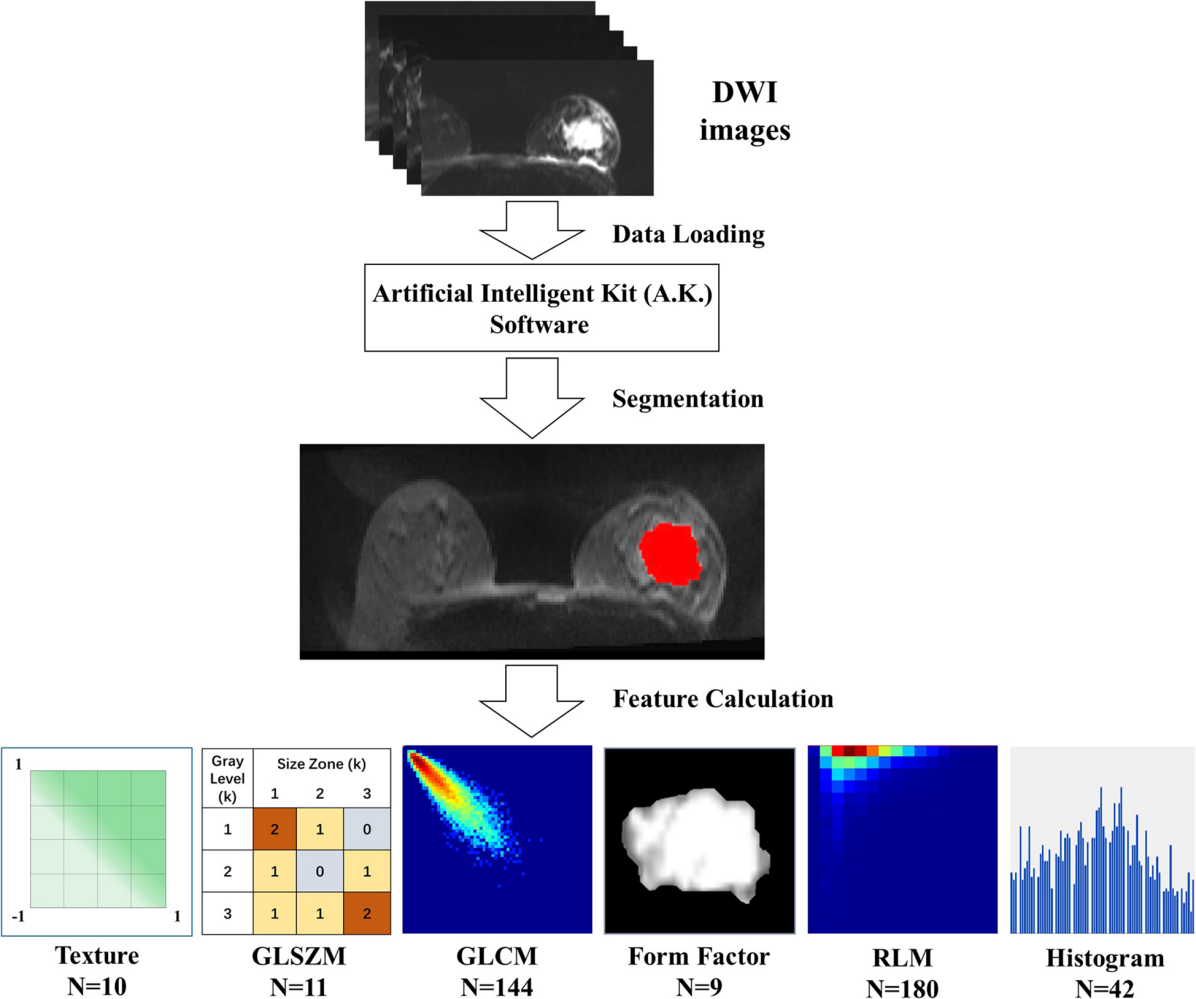
Workflow of Segmentation and Extraction of radiomic features

A total of 396 radiomic features could be derived from the VOI of the DW image by A.K. software, as shown in Fig. 1. These features were categorized into six statistical methods, including texture parameters, grey level size zone matrix (GLSZM), grey level co-occurrence matrix (GLCM), form factor parameters, run length matrix (RLM) and histogram. Texture parameters represent the appearance of the surface and how its elements are distributed. GLSZM provides a statistical representation by the estimation of a bivariate conditional probability density function of the image distribution values. GLCM represents the joint probability of certain sets of pixels with certain grey-level values. RLM is defined as the number of runs with pixels of grey-level *i* and run length *j* for a given direction θ.

### Pre-processing

**Fig. 2:**
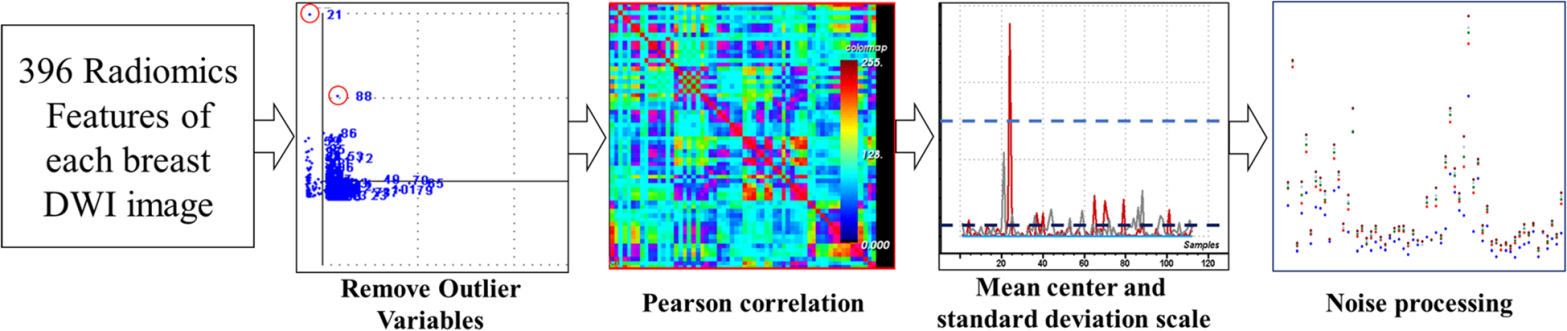
Preprocessment of 396 radiomic features

The training dataset was built by 396 radiomic parameters from 112 breast cancer cases. To eliminate redundant radiomic parameters, the pre-processing of the training dataset was performed as follows (Fig. 2). First, if one value of a certain radiomic feature was out of the range of the mean ± standard deviation (SD), it would be considered an outlier and then removed from the dataset. Second, Pearson correlation analysis was conducted on two radiomic features in the training dataset, and if the correlation coefficient between pairwise features was above 0.9, one of the two features would be removed by random selection. Third, the mean centre and standard deviation scale were used to standardize the variables to the same value range. Finally, noise processing with linear smoothing filtering was automatically performed by A.K. software [28, 29].

### Classifier building

Fisher discriminant analysis was used for clinicopathological subtyping by using a backward selection method [30]. An approach of 104 iterations and 84 variables were used to establish the Fisher discriminant model (Function 1 to Function 5).

To illustrate the process of building the Fisher discriminant model for the differential analysis of the five clinicopathological subtypes of breast cancer, the equations shown below were used, where X_i_ was the radiomic feature used for the function building, and Y_i_ was the class of one specified unbeknown patient. To ensure the accuracy of the model, we used the whole data set to calculate functions of Fisher discriminant model. Fisher discriminant model was trained by (n-1) samples and validated by the remaining sample.

$$ {Y}_1=143.07{X}_1+132.35{X}_2+\dots +153.22{X}_{83}-58.30{X}_{84}-1273.19 $$

$$ {Y}_2=180.48{X}_1+163.90{X}_2+\dots +180.05{X}_{83}-96.49{X}_{84}-1668.82 $$

$$ {Y}_3=168.72{X}_1+138.75{X}_2+\dots +209{X}_{83}+7.66{X}_{84}-1630.72 $$

$$ {Y}_4=232.37{X}_1+198.77{X}_2+\dots +241.92{X}_{83}-76.65{X}_{84}-2236.77 $$

$$ {Y}_5=187.87{X}_1+143.57{X}_2+\dots +185.89{X}_{83}-37.95{X}_{84}-1505.12 $$

The leave-one-out cross-validation method was used for testing the Fisher discriminant analysis model. If the sample size was *n*, leave-one-out cross-validation was accomplished by the prediction of the remaining samples with the discriminant model established by *n-1* samples, and the final prediction results for all samples would be obtained after the iterations (*n* times) and then was regarded as the criteria standard for the prediction of the model [31].

To overcome the shortcoming of data imbalance, the mean class-weighted accuracy (CWA) was performed following the method proposed by Cohen et al. [32]. The equation for k-class setting is as follow:

$$ cwa=\frac{1}{\sum_{i=1}^k{w}_i}{\sum}_{i=1}^k{w}_i{accu}_i $$

Where *w*_*i*_ is the weight assigned to class *i* and *accu*_*i*_ is the accuracy rate computed over class *i*.

### Predicting different statuses of IHC biomarkers

To predict the different statuses of immunohistochemical biomarkers, the diagnostic performance of radiomic features was assessed by receiver operating characteristic (ROC) curve analysis with a two-step approach. Radiomic features after pre-processing were included to calculate the predicted value of ER status, PR status, HER2 status and Ki-67 index by binary logistic regression. Then, ROC analysis was performed by using those predicted values. Based on the predicted values, ROC analyses were performed to differentiate between the ER positive and negative group, PR positive and negative group, HER2 positive and negative group, and Ki-67 low and high group. The comparison of the areas under two ROC curves was calculated by MedCalc software (Version 11.4.2, Mariakerke, Belgium) following the methodology of DeLong et al. [33].

Binary logistic regression and ROC analyses were performed using IBM SPSS software version 19.0 (IBM Corporation, New York). A *p*-value < 0.05 was considered statistically significant.

## Results

There were 29 luminal A cases, 31 luminal B_HER-_ cases, 17 luminal B_HER+_ cases, 24 HER2-positive cases and 11 triple-negative cases in our study (Table 2).

**Table 2:** General features and clinicopathological subtypes

Characteristic	Patients (***n*** = 112)	Clinicopathologic subtypes				
		Luminal A	Luminal B_HER2-_	Luminal B _HER2+_	HER2 positive	Triple negative
Age	46.5(25 ~ 72)	46.1 (25 ~ 69)	47.4 (32 ~ 67)	44.6 (26 ~ 69)	45.8 (28 ~ 72)	49.5 (30 ~ 61)
ER status						
Positive	75	29	29	17	0	0
Negative	37	0	2	0	24	11
PR status						
Positive	69	27	29	13	0	0
Negative	43	2	2	4	24	11
HER2 status						
Positive	41	0	0	17	24	0
Negative	71	29	31	0	0	11
Ki-67						
≥ 14%	81	0	31	16	23	11
< 14%	31	29	0	1	1	0

A total of 162 radiomic features of four statistical methods were included after preprocessing. Of the 162 radiomic features, there were 42 histogram parameters, 18 texture parameters, 52 GLCM parameters and 50 RLM parameters. Each of 162 features would be evaluate in the Fisher discriminant analysis to determine its contribution, with which feature contributed to modelling would be held. Finally, 84 features were used for the model-building.

The overall accuracy for predicting the clinicopathological subtypes was 96.4% by Fisher discriminant analysis. Based on class-weighted accuracy calculating, the overall weighted accuracy was 96.6%. For predicting diverse clinicopathological subtypes, the prediction accuracies ranged from 92 to 100%. When predicting subtypes of luminal B_HER2-_ and triple negative, both accuracies were 100% (Table 3).

**Table 3:** Fisher discriminant analysis and cross-validation

	Subtypes/n	Training dataset					Prediction accuracy
		1	2	3	4	5	
Fisher discriminant analysis	1	28	0	1	0	0	97%
	2	0	31	0	0	0	100%
	3	0	0	16	0	1	94%
	4	1	1	0	22	0	92%
	5	0	0	0	0	11	100%
Total	–	–	–	–	–	–	96.4%
Leave-one- out cross- validation	1	23	4	0	0	2	79%
	2	1	24	0	1	5	77%
	3	1	0	15	1	0	88%
	4	0	2	0	22	0	92%
	5	3	0	0	0	8	73%
Total	–	–	–	–	–	–	82.1%

Subtypes: 1, luminal A; 2, luminal B_HER2-_; 3, luminal B_Her2+_; 4, HER2 positive; 5, triple negative

A leave-one-out cross-validation was performed to test the Fisher discriminant analysis model. The overall accuracy of the model was 82.1% in the prediction of the clinicopathological subtypes of breast cancer. The accuracies of the model for predicting the luminal A, luminal B_Her2-_, luminal B_Her2+_, Her2-positive and triple-negative subtypes were 79, 77, 88, 92 and 73%, respectively (Table 3).

**Fig. 3:**
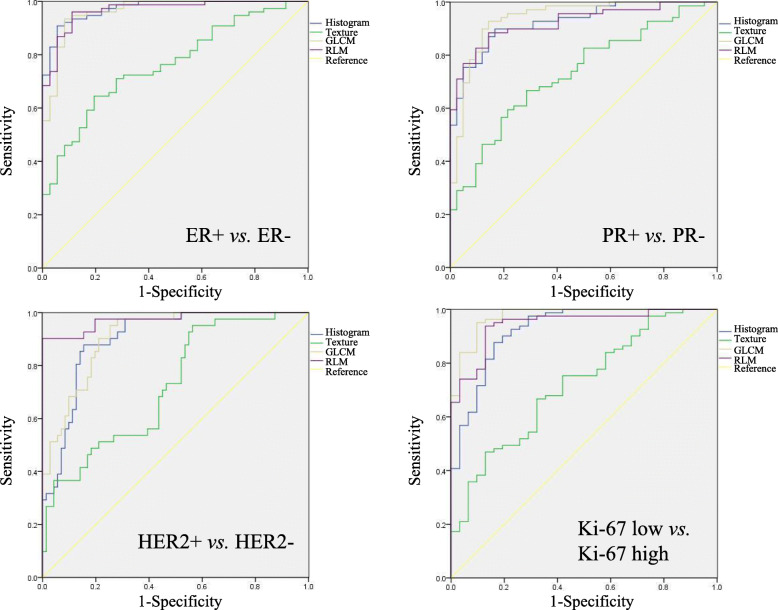
ROC analysis of radiomic features. AUROCs of histogram parameters, GLCM parameters and RLM parameters were higher than those of texture parameters in assessing status of ER, PR, HER2 and Ki-67 (*p* < 0.001)

The areas under the ROC curve (AUROCs) of histogram parameters, texture parameters, GLCM parameters and RLM parameters for predicting different IHC biomarkers are shown in detail in Table 4 and Fig. 3. Furthermore, the AUROCs of histogram parameters, GLCM parameters and RLM parameters were higher than those of texture parameters in assessing the status of ER, PR, HER2 and Ki-67 (*p* < 0.001) (Table 5).

**Table 4:** ROC analysis of radiomic features in prediction of immunohistochemical status

IHC status	Radiomic feature	AUROC
**ER(+) VS. ER(−)**	Histogram	0.973 (0.949–0.997)
	Texture	0.762 (0.674–0.851)
	GLCM	0.963 (0.929–0.998)
	RLM	0.967 (0.937–0.997)
**PR(+) VS. PR(−)**	Histogram	0.925 (0.879–0.972)
	Texture	0.731 (0.637–0.824)
	GLCM	0.939 (0.892–0.986)
	RLM	0.923 (0.875–0.971)
**HER2(+) VS. HER2(−)**	Histogram	0.902 (0.847–0.957)
	Texture	0.722 (0.627–0.818)
	GLCM	0.911 (0.860–0.962)
	RLM	0.974 (0.944–1.000)
**Ki-67 low VS. high**	Histogram	0.926 (0.870–0.981)
	Texture	0.718 (0.615–0.820)
	GLCM	0.975 (0.949–1.000)
	RLM	0.946 (0.905–0.988)

**Table 5:** Comparison of ROC analysis results

IHC status	AUROC	Z statistic	***p*** value
**ER(+) VS. ER(−)**	Histogram VS. Texture	4.531	< 0.001
	Histogram VS. GLCM	0.462	0.644
	Histogram VS. RLM	0.312	0.7548
	Texture VS. GLCM	−4.147	< 0.001
	Texture VS. RLM	−4.322	< 0.001
	GLCM VS. RLM	−0.171	0.864
**PR(+) VS. PR(−)**	Histogram VS. Texture	3.676	< 0.001
	Histogram VS. GLCM	−0.412	0.680
	Histogram VS. RLM	0.058	0.954
	Texture VS. GLCM	−3.941	< 0.001
	Texture VS. RLM	−3.607	< 0.001
	GLCM VS. RLM	0.462	0.644
**HER2(+) VS. HER2(−)**	Histogram VS. Texture	3.189	0.001
	Histogram VS. GLCM	−0.236	0.814
	Histogram VS. RLM	−2.267	0.023
	Texture VS. GLCM	−3.407	< 0.001
	Texture VS. RLM	−4.918	< 0.001
	GLCM VS. RLM	−2.099	0.036
**Ki-67 low VS. high**	Histogram VS. Texture	3.493	< 0.001
	Histogram VS. GLCM	−1.542	0.123
	Histogram VS. RLM	−0.559	0.576
	Texture VS. GLCM	−4.795	< 0.001
	Texture VS. RLM	−4.066	< 0.001
	GLCM VS. RLM	1.174	0.240

## Discussion

Our study has shown that the Fisher discriminant analysis model with radiomic features of DW images can be used for predicting the clinicopathological subtypes of breast cancer. Furthermore, the model had excellent accuracies ranging from 92 to 100% in the prediction of clinicopathological subtypes. Based on the leave-one-out cross-validation, the overall accuracy was 82.1% when testing the Fisher discriminant analysis model.

In our study, each clinicopathological subtype could be distinguished from others with high accuracy. Furthermore, we applied Fisher’s discriminant analysis to resolve a multiple classification problem, i.e., five clinicopathological subtypes of breast cancer. The clinicopathological subtypes of breast cancer are defined according to their therapeutic purposes. Our findings may contribute to decision-making and treatment selection in clinical practice, such as endocrine therapy, cytotoxic therapy, and anti-HER2 therapy [5]. As mentioned above, the molecular luminal B subtype is divided into two new clinicopathological subtypes, luminal B with or without HER2 overexpression (i.e., luminal B_HER2-_ and luminal B_HER2+_). Luminal B_HER2+_ cases require cytotoxics, endocrine therapy and anti-HER2 therapy, whereas a luminal B_HER2-_ cases does not require anti-HER2 therapy. Therefore, the clinicopathological subtype can provide detailed therapeutic information. Moreover, compared with molecular subtypes from gene assays, the clinicopathological subtypes from immunohistochemistry can be easily obtained with lower costs.

In addition, leave-one-out cross-validation was performed to evaluate the accuracy of the Fisher discriminant analysis model. Our results showed that the overall accuracy of the model was 82.1% in predicting the clinicopathological subtypes. The accuracy for predicting HER2 positivity was up to 92%. We confirmed that the Fisher discriminant analysis model is a reliable method that can be used for predicting the clinicopathological subtypes of breast cancer. More importantly, our study validated the feasibility of using the Fisher discriminant analysis model to settle a multi-classification problem.

In our study, we provided a noninvasive method for the prediction of the clinicopathological subtypes of breast cancer by using radiomic features of DWI. DWI is a widely used method that can measure the Brownian motion of water molecules. The motion of water molecules in tissue can be affected by tissue cellularity and membrane integrity [6]. DWI has been applied in the detection of breast cancer [34], differentiation of benign and malignant lesions [35], and monitoring the response to neoadjuvant chemotherapy [36, 37]. However, there is no consensus that DWI is a reliable stand-alone method. One recent study showed that the combination of T2-weighted fat suppression and DWI textural features could predict sentinel lymph node metastasis [38]. By using diffusion MRI, a radiomic signature was shown to differentiate malignant from benign lesions [39]. Furthermore, another study showed that ADC values correlate with the biological features of breast cancer [40]. In addition to DWI mentioned above, a number of studies have focused on radiomic analysis from DCE-MRI. The imaging features from DCE-MRI can predict the luminal A and luminal B molecular subtypes [24] and can also differentiate between the histological and immunohistochemical subtypes of breast cancer [41]. One DCE-MRI feature that quantifies the relationship between lesion enhancement and background parenchymal enhancement is associated with the luminal B subtype of breast cancer [42]. However, these imaging feature-based studies provide insufficient information for the differential diagnosis of the five clinicopathological subtypes of breast cancer. To resolve this problem, our study focused on the prediction of clinicopathological subtypes by using DW imaging features and showed a high diagnostic performance with an overall accuracy of 96.4%.

For predicting breast cancer receptor status and molecular subtyping, Leithner et al. showed that the breast tumour segmentation approaches could affect the classification accuracy by using radiomic signature of DWI [43]. In their study, two segmentation approaches included: (1) segmentation ROI performed on high b value DWI and copied to ADC map; (2) segmentation ROI drawn directly on ADC map. The results indicated that tumour segmentation directly on ADC map was of better classification accuracy. However, in their study, some lesions could not be identified on ADC map. Our study only drawn segmentation ROI on DWI without ADC map, on which distinct tumour margin could easily be confirmed.

The multiparametric MR radiomics using DCE and DWI can also be performed for predicting breast cancer subtypes [44]. Text features were extracted form DCE images of six contrast-enhanced phases and DWI with three b-values. The best accuracies of multiparametric MR radiomics model were 72.4 and 91.0% for the 4-IHC classification task and for the TN vs. non-TN cancers, respectively. However, for 4-IHC classification task, the accuracy of DWI with linear discriminant analysis model was 53.7% by using minor dependence emphasis on Kendall-tau-b. And for differentiating triple negative (TN) from non-TN tumours, the accuracy of DWI was 83.6% by using the maximum of variance. It indicated that the multiparametric MR radiomics performed well than DWI for 4-IHC classification task and for the TN vs. non-TN cancers.

Recently, Leithner et al. apply artificial intelligence (AI) to breast cancer molecular subtyping with multiparametric MR radiomics [45]. Texture features extracted from DCE images and ADC maps and a multi-layer perceptron feed-forward artificial neural network (MLP-ANN) were used for differentiation of TN and luminal A breast cancers from other subtypes. Their results indicated that multiparametric MR radiomics could provide prognostic and predictive information derived from the entire tumour before and during treatment.

In predicting the different statuses of IHC biomarkers, excellent diagnostic performance of radiomic features was found in differentiating between the ER-positive and -negative group, PR-positive and -negative group, HER2-positive and -negative group, and Ki-67-low and -high group. However, there were relatively low AUROCs of texture parameters in assessing the status of these biomarkers. This result may be ascribed to a small number of 18 texture parameters. Compared with our results, one study using 38 radiomic features showed a less powerful result, with AUROCs ranging from 0.641 to 0.789 in predicting ER, PR and HER2 statuses [46]. Another study showed a similar result with AUROCs ranging from 0.65 to 0.89 [14]. Therefore, radiomics-based approach can serve as a non-invasive method for predicting breast cancer receptor status.

In our study, histogram, GLCM and RLM features had high diagnostic performance in differentiating different IHC biomarkers. For differentiating ER-positive and ER-negative cases, PR-positive and PR-negative cases, HER2-positive and HER2-negative cases, and Ki-67-low and Ki-67-high cases, the AUROCs of histogram, GLCM and RLM features were 0.963–973, 0.923–0.939, 0.902–0.974 and 0.926–0.975, respectively. Furthermore, the Fisher discriminant analysis model had high accuracy in predicting the five clinicopathological subtypes of breast cancer. We concluded that radiomic features of DWI were highly associated with IHC biomarkers and could be emerging surrogates for IHC biomarkers. With the Fisher discriminant analysis model, we provided a new radiomic approach for breast cancer subtyping, and its findings contribute to treatment selection.

Our study has some limitations. First, the main limitations of the study are its small sample size and lack of independent external validation, which makes the results difficult to reproduce in other populations. We will enlarge the sample size and apply other methods for the clinicopathological classification of breast cancer. Second, the automatic segmentation method was not applied in our study. Hence, the ROI delineation was time-consuming. Third, the apparent diffusion coefficient (ADC) value for each breast tumour was not acquired. Therefore, we failed to compare the diagnostic performance of ADC with that of radiomic features. Fourth, the lack of morphological features could limit our results. Our future work will focus on resolving these limitations.

## Conclusions

Our study demonstrated that the Fisher discriminant analysis model based on radiomic features of diffusion-weighted MRI could be used for the prediction of the clinicopathological subtypes of breast cancer with high accuracy. Moreover, the radiomic features had excellent diagnostic performance in differentiating between the ER-positive and -negative groups, PR-positive and -negative groups, HER2-positive and -negative groups, and Ki-67-low and -high groups. More breast cancer cases will be enrolled in our future work to validate this radiomic approach.

## Data Availability

The datasets used and/or analysed during the current study are available from the corresponding author on reasonable request.

## Abbreviations

HER2: Human epidermal growth factor receptor 2
IHC: Immunohistochemical
ER: Oestrogen receptor
PR: Progesterone receptor
DWI: Diffusion-weighted imaging
MRI: Magnetic resonance imaging
DCE-MRI: Dynamic contrast-enhanced MRI
PACS: Picture archiving and communication system
ROI: Region of interest
VOI: Volume of interest
GLSZM: Grey level size zone matrix
GLCM: Grey level co-occurrence matrix
RLM: Run length matrix
SD: Standard deviation
CWA: Class-weighted accuracy
ROC curve: Receiver operating characteristic curve
ADC: Apparent diffusion coefficient
